# Diagnostic value of neutrophil-to-lymphocyte ratio in patients with leprosy reactions

**DOI:** 10.1016/j.heliyon.2020.e03369

**Published:** 2020-02-13

**Authors:** Luciano Teixeira Gomes, Yvelise Terezinha Morato-Conceição, Ana Vitória Mota Gambati, Carolina Mira Maciel-Pereira, Cor Jesus Fernandes Fontes

**Affiliations:** a Júlio Müller University Hospital, Department of Infectious Diseases, Faculty of Medicine, Federal University of Mato Grosso, Cuiabá, Mato Grosso, Brazil; b Faculty of Biomedical Sciences of Cacoal, Cacoal, Rondonia, Brazil

**Keywords:** Health sciences, Infectious disease, Medical microbiology, Clinical research, Diagnostics, Biomarkers, Leprosy, Leprosy reaction, Neutrophil-to-lymphocyte ratio, Diagnosis, Biomarker

## Abstract

**Introduction:**

Leprosy reactions, classified as type 1 and type 2 reactions, are acute clinical conditions of exacerbation of localized or systemic inflammatory response inpatients with leprosy. No laboratory biomarker is available to predict the emergence of these reactions. Neutrophil-to-lymphocyte ratio (NLR) is an accurate biomarker for diagnosis and prognosis of various inflammatory and neoplastic diseases.

**Objective:**

This study aimed to investigate the accuracy of the NLR in the diagnosis of leprosy reactions.

**Materials and methods:**

NLR was calculated for all patients and a receiver operating characteristic curve (ROC) were generated to identify the NLR cut-off point.

**Results:**

A total of 123 patients with leprosy were included, 98 with leprosy reactions of which 56 (45.5%) had type 1 and 42 (34.1%) with type 2. Mean NLR was higher among patients with reactions than among those without. It was also statistically higher among patients with type 2 reactions than in those with type 1 reactions. Receiver operating characteristic curves were generated to identify the NLR cut-off point. The area under the ROC curve was 0.794 for diagnosis of any leprosy reaction and 0.796 for the diagnosis of type 2 reaction. The NLR cut-off points for diagnosis of any leprosy reaction and for type 2 reaction were 2.75 (sensitivity 61.0%, specificity 92.0%, accuracy 77.0%) and 2.95 (sensitivity 81.0%, specificity 74.0%, accuracy 78.0%), respectively.

**Conclusion:**

These results suggest that NLR could be a potential biomarker for diagnosis of leprosy reaction and useful for discriminating patients with type 2 reactions from those with type 1 leprosy reactions.

## 1 Introduction

Leprosy is a chronic granulomatous infectious disease caused by *Mycobacterium leprae*, which mainly affects the skin and peripheral nervous system. If not recognized, the infection can lead to permanent nerve damage and progressive disability. The disease is hyperendemic in Africa, South America, and Asia; with increasing migrations around the world, new cases have been increasingly detected in developed countries (WHO, 2016).

According to World Health Organization (WHO) guidelines, patients with five or fewer skin lesions generally do not have bacilli in skin smears and are classified as paucibacillary. Those with five or more skin lesions may have positive smear microscopy and therefore are classified as multibacillary (WHO, 2012). Cellular immunity plays a relevant role in disease evolution. Lymphocytes from paucibacillary patients demonstrate appropriate function against *M. leprae*, proliferating in response to bacillus antigens. In contrast, multibacillary patients do not exhibit proliferation against these antigenic stimuli and thus are not able to eliminate the bacilli (Nath et al., 2015). Based on cutaneous findings, motor alterations, sensory alterations, and histologic alterations, leprosy can be classified more precisely as indeterminate, tuberculoid, borderline, and lepromatous. A strong cell-mediated immune response and weak humoral response characterize the immunity of the tuberculoid form of the disease. The opposite is observed in the lepromatous form (Jacobson and Krahenbuhl, 1999).

Leprosy reactions, which can be classified as type 1 or type 2 reactions, are acute clinical conditions of exacerbation of localized or systemic inflammatory response in patients with leprosy. Type 1 reactions are caused by activation of the cellular immune response to *M. leprae* antigens. Clinically, there is increased inflammation of pre-existing skin lesions and intense neuritis, which can lead to impairment of sensory and motor neurons of the affected region. Systemic symptoms are rare, and visceral impairment does not occur. Treatment of type 1 reactions is mainly accomplished with high doses of prednisone (Raffe et al., 2013). On the other hand, type 2 leprosy reactions, or erythema nodosum leprosum, involve acute inflammation of the dermal vessels as well as acute inflammation of subcutaneous and other tissues invaded by the bacilli. The typical clinical sign of this reaction is the sudden onset of a large number of painful erythematous nodules throughout the body. The recurrence of type 2 reactions is frequent and may persist for years (Scollard et al., 2006). Laboratory findings associated with type 2 reactions comprehend increased acute phase proteins, including C-reactive protein (CRP), α1-antitrypsin (Kahawita and Lockwood, 2008), α1-acid glycoprotein (Gupta et al., 2010) and gamma globulins (Foss and Motta, 2012). The treatment of type 2 reactions consists of administration of corticosteroid, pentoxifylline, or thalidomide (Raffe et al., 2013).

With the objective of eliminating leprosy in the world, the World Health Organization recently presented actions to reduce leprosy-induced deficiencies and sequelae, mainly directed against leprosy reactions (WHO, 2016). The immediate diagnosis of the reactional episodes significantly contributes to recovery of the patient, reducing the risks of permanent tissue damage (Lockwood and Saunderson, 2012). Unfortunately, these reactions are often diagnosed late because of unprepared health services (Raffe et al., 2013). Therefore, reliable tests for the early diagnosis of leprosy reactions can make a big difference in the clinical outcomes of the disease. However, a major obstacle to development of such tests is the lack of reliable biomarkers associated with type 1 and type 2 reactions in patients with leprosy in endemic areas.

The total leukocyte count can increase dramatically in response to infections, trauma, unhealthy diet, and chronic physiological or psychological stress (Riley and Rupert, 2015). Neutrophils are leukocytes that act in the early phase of leprosy, by phagocytosis of the bacillus and release of pro-inflammatory mediators. Among the various parameters related to blood leukocytes, the neutrophil-to-lymphocyte ratio (NLR) stands out and is significantly associated with the systemic inflammation (Papa et al., 2008; Benites-Zapata et al., 2015). This was first studied in 1995 in patients with acute appendicitis (Goodman et al., 1995) and has been recently reemphasized (de Jager et al., 2012). In fact, several studies concerning NLR have been published in the last 3 years, highlighting its importance as an independent prognostic factor for solid tumor (Templeton et al., 2014) and as an inflammatory biomarker in several acute and chronic cardiovascular and metabolic diseases (Bhat et al., 2013). As NLR combines neutrophils and lymphocytes in its calculation, it is considered more stable than absolute neutrophil or lymphocyte counts (Dirican et al., 2015). NLR is accepted as a parameter that reflects the negative effects of high numbers of neutrophils that indicate an acute inflammatory response. Beside this, NLR also reflects the effects of low numbers of lymphocytes that indicate the simultaneous deterioration of general health and physiological stress (Yamanaka et al., 2007).

NLR has been studied in several inflammatory states and neoplastic diseases including ulcerative colitis (Torun et al., 2012), acute pancreatitis (Azab et al., 2011), breast cancer (Azab et al., 2012), lung cancer (Cedrés et al., 2012), and hepatocellular carcinoma (Gomez et al., 2008). As for infections, NLR is a predictor of the severity and clinical outcome in patients with community-acquired pneumonia (de Jager et al., 2012) or bacteremia (Loonen et al., 2014).

Identification of accurate biomarkers for diagnosis and follow-up of patients with chronic diseases, including leprosy, will enable improved evaluation of their clinical characteristics and the diagnosis of those more susceptible to complications. NLR is a biomarker that is easily obtained and available in clinical practice. The objective of this study was to describe NLR values in patients with leprosy and to evaluate the role of NLR in the diagnosis of patients with leprosy reactions of types 1 and 2. To the best of the authors’ knowledge, no research on NLR related to diagnosis of leprosy reactions has yet been conducted.

## 2 Materials and methods

This was a cross-sectional descriptive study of data obtained from medical records of patients seen between June 2012 and August 2015 at the Reference Centre for Tropical Diseases of the Júlio Müller University Hospital, Cuiabá, Brazil. Eligible patients were aged between 18 and 69 years and had a confirmed diagnosis of leprosy based on dermato-neurological or laboratory examination. Patients with other mycobacteriosis, autoimmune diseases, neoplasms, diabetes mellitus, or evidence of any type of immunodepression, such as HIV/AIDS or use of immunosuppressive drugs, were excluded from the analysis. Some patients had already completed treatment for leprosy, whereas others were still being treated or had started treatment after being admitted. This study was approved by the Research Ethics Committee of the Júlio Müller University Hospital, under approval number 19502.

Diagnosis of the leprosy reaction was made by physicians experienced in the diagnosis and treatment of leprosy and was confirmed by a second specialist physician. Patients were classified into three groups: type 1 leprosy reaction (R1), type 2 leprosy reaction (R2), and no leprosy reaction (NR). Eyes, hands, and feet were checked for symptoms and deformities. For sensory testing a nylon filament was used on the hands and feet to test protective sensibility. Disability grading was done according to the WHO-2-point scale (WHO, 2012). For these patients, we recorded the following laboratory results: blood cell count, erythrocyte sedimentation rate, serum lipid levels, liver enzymes, glucose, urea, creatinine, lactic dehydrogenase, C-reactive protein, and α1-acid glycoprotein. The overall WBC count was determined for all of the samples using automated blood cell counting equipment (Sysmex XE-2100D, Kobe, Japan). NLR was calculated for all patients.

Treatment of the leprosy reaction and collection of blood to determine baseline values was initiated only after admission to the study. All patients with type 1 reactions received oral prednisone (40–80 mg/day for 4 weeks), and then the prednisone was gradually reduced. Type 2 reactions were treated with thalidomide, 100–400 mg/day, except when contraindicated. In type 2 reactions, prednisone has been associated with concomitant neuritis or necrotic erythema nodosum (Raffe et al., 2013).

A descriptive analysis was initially performed for all study variables. Values are reported as mean ± standard deviation. The Shapiro-Wilk test was used to verify normality for the distribution of the biochemical and hematological results. Since all analyzed parameters showed non-normal distributions, the non-parametric Mann-Whitney test was used to compare continuous variables between groups. Proportions were compared using Pearson's chi-square test. A receiver operator characteristic (ROC) curve was constructed to evaluate the sensitivity and specificity of NLR for diagnosis of leprosy reactions, as well as the type of leprosy reaction. The area under the curve (AUC) was calculated; higher values indicate greater discriminatory capacity of the leprosy reaction type. Youden's Index J was calculated to define empirical cut-offs corresponding to sensitivity and specificity values less likely to have occurred at random (Bantis et al., 2014). The AUC confidence interval (CI) was calculated using a non-parametric assumption. A value of p < 0.05 was considered to represent a statistically significant difference. Statistical analyses were performed using Stata software (Stata Corporation, College Station, TX, USA).

## 3 Results

One hundred twenty-three leprosy patients sought treatment at the reference center during the study period; 25 (20.3%) of whom had tuberculoid leprosy, 56 (45.5%) had borderline leprosy, and 42 (34.2%) had lepromatous leprosy. Thirteen patients (10.6%) were classified as having paucibacillary leprosy, and 110 (89.4%) as having multibacillary leprosy. A skin lymph smear examination was performed in 104 (84.6%) patients and was positive in 87 (83.7%) of them. In multibacillary patients, the bacilloscopic index ranged from 0.25 to 9.0, and it was equal to or greater than 3.0 in 58 (55.8%) patients (Table 1). The majority of the patients were male (74.0%), with a mean age of 41.4 ± 12.6 years. Comorbidities diagnosed in these patients after inclusion in the study included systemic arterial hypertension (8.9%), smoking (19.5%), and hyperlipidemia (5.7%). The evaluation exam for disability at the beginning of treatment showed some degree of functional impairment in 53 (43.1%) patients (Table 1).

**Table 1:** Demographic and clinical characteristics of the 123 patients in the study, according to presence and type of leprosy reaction.

Characteristics		n (%)	Leprosy reaction		p*	Leprosy reaction Type 1		p*	Leprosy reaction Type 2		p*
			Present	Absent		Present	Absent		Present	Absent	
Clinical form	*Tuberculoid*	25 (20.3)	0 (0.0)	25 (100.0)	<0.001	0 (0.0)	25 (100.0)	<0.001	0 (0.0)	25 (100.0)	<0.001
	*Borderline*	56 (45.5)	56 (100.0)	0 (0.0)		56 (100.0)	0 (0.0)		0 (0.0)	56 (100.0)	
	*Lepromatous*	42 (44.2)	42 (100.0)	0 (0.0)		0 (0.0)	42 (100.0)		42 (100.0)	0 (0.0)	
WHO Classification	*Paucibacillary*	13 (10.6)	0 (0.0)	13 (100.0)	<0.001	0 (0.0)	13 (100.0)	<0.001	0 (0.0)	13 (100.0)	0.006
	*Multibacillary*	110 (89.4)	98 (89.1)	12 (10.9)		56 (50.9)	54 49.1)		42 (38.2)	68 (61.8)	
Microscopy	*Negative*	17 (16.4)	12 (70.6)	5 (29.4)	0.012	12 (70.6)	5 (29.4)	0.052	0 (0.0)	17 (100.0)	<0.001
	*Positive*	87 (83.6)	80 (92.0)	7 (8.0)		39 (44.8)	48 (55.2)		41 (47.1)	46 (52.9)	
Bacilloscopic Index∗	*< 3.0*	46 (44.2)	27 (93.1)	2 (6.9)	0.780	20 (69.0)	9 (31.0)	0.001	7 (24.1)	22 (75.9)	0.002
	*≥ 3.0*	58 (55.8)	53 (91.4)	5 (8.6)		19 (32.8)	39 (67.2)		34 (58.6)	24 (41.4)	
Sex	*Male*	91 (26.0)	77 (84.6)	14 (15.4)	0.022	12 (37.5)	20 (62.5)	0.289	9 (28.1)	23 (71.9)	0.404
	*Female*	32 (74.0)	21 (65.6)	11 (34.4)		44 (48.4)	47 (51.6)		33 (36.3)	58 (63.7)	
Color	*White*	52 (42.3)	39 (75.0)	13 (25.0)	0.270	25 (48.1)	27 (51.9)	0.627	14 (26.9)	38 (73.1)	0.148
	*Non white*	71 (57.7)	59 (83.1)	12 (16.9)		31 (43.7)	40 (56.3)		28 (39.4)	43 (60.6)	
Age (years)	*18–30*	29 (23.6)	23 (79.3)	6 (20.7)	0.998	10 (34.5)	19 (65.5)	0.099	13 (44.8)	16 (55.2)	0.089
	*31–50*	59 (48.0)	47 (79.7)	12 (20.3)		25 (42.4)	34 (57.6)		22 (37.3)	37 (62.7)	
	*> 50*	45 (28.4)	28 (80.0)	17 (20.0)		21 (60.0)	14 (40.0)		7 (20.0)	28 (80.0)	
MDT Treatment	*New*	37 (30.1)	24 (64.9)	13 (35.1)	<0.001	20 (54.0)	17 (46.0)	0.153	4 (10.8)	33 (80.2)	<0.001
	*Current*	45 (36.6)	33 (73.3)	12 (26.7)		23 (51.1)	22 (48.9)		28 (62.2)	17 (37.8)	
	*Previous*	41 (33.3)	41 (100.0)	0 (0.0)		14 (34.1)	27 (65.9)		10 (24.4)	31 (75.6)	

∗ n = 87; WHO: World Health Organization; MDT: multi-drug therapy for leprosy. *: Pearson´s chi-square test.

At the initial evaluation, 98 (79.7%) patients presented leprosy reactions, 56 (45.5%) with type 1 and 42 (34.1%) with type 2. MDT was started after inclusion in the study for 37 patients: 13 with no reaction, 20 with type 1 reaction, and 4 with type 2 reaction. Forty-one (32.3%) patients had already completed leprosy treatment (28 with type 2 reactions and 13 with type 1 reactions) and 45 were still on MDT (12 with no reaction, 23 with type 1 reactions, and 10 with type 2 reactions). Leprosy reactions were more frequent in patients with borderline and lepromatous leprosy, in patients with positive bacilloscopy, in patients with a bacilloscopic index ≥3, and in patients who had received treatment or were being treated for leprosy at the time of inclusion in the study (Table 1).

**Figure 1:**
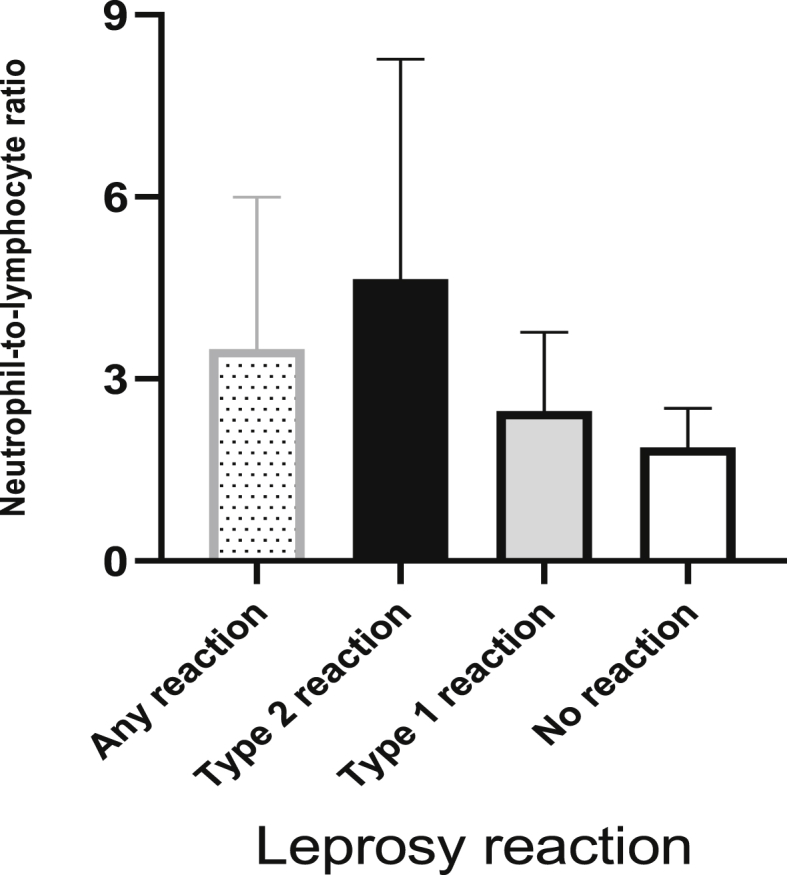
Neutrophil-to-lymphocyte ratio of patients with leprosy, according to presence or absence of leprosy reaction.

Mean neutrophil count was significantly higher among patients with any leprosy reaction, regardless of whether they had type 1 or type 2 reactions, as well as those with multibacillary leprosy and those with a bacilloscopic index ≥3. However, no association was observed between lymphocyte count and leprosy reactions. In contrast, mean NLR was higher (p < 0.001) among patients with leprosy reactions (6.1 ± 10.8) than among those without reactions (2.0 ± 0.7). Mean NLR was also higher among patients with a type 2 reaction (9.7 ± 2.4; p < 0.001) than among those with a type 1 or with no leprosy reaction (3.4 ± 2.5). Lower NLR were observed among patients with type 1 or with no leprosy reaction (Table 2, Figure 1).

**Table 2:** Blood count of neutrophils and lymphocytes and neutrophil ratio for lymphocytes among leprosy patients, according to clinical characteristics of patients with leprosy.

Leprosy reaction		Neutrophils (SD)	p∗	Lymphocytes (SD)	p∗	NLR (SD)	p∗
Any	*Present*	8,108 (5,921)	<0.001	1,918 (948)	0.254	6.1 (10.8)	<0.001
	*Absent*	2,730 (1,295)		1957 (544)		2.0 (0.7)	
Type 1	*Present*	5,490 (3,628)	0.003	1,778 (677)	0.078	3.4 (2.5)	0.144
	*Absent*	8,662 (6,504)		2,049 (1,006)		6.8 (12.9)	
Type 2	*Present*	11,598 (6,592)	<0.001	2,104 (1,204)	0.377	9.7 (15.6)	<0.001
	*Absent*	4,947 (3,197)		1,833 (641)		3.0 (2.2)	
WHO Classification	*Multibacillary*	7,595 (5,784)	0.013	1,906 (910)	0.105	5.7 (10.2)	<0.001
	*Paucibacillary*	4,028 (1,554)		2,095 (545)		2.0 (0.8)	
Bacilloscopic Index (n = 87)	*< 3.0*	5,914 (4,041)	0.001	1,925 (845)	0.588	5.3 (14.2)	0.114
	*≥ 3.0*	9,122 (6,607)		1,885 (972)		6.2 (6.3)	

Values shown mean (standard deviation).

∗ Non-parametric Mann–Whitney test.

For patients with multibacillary leprosy, mean NLR was 5.7 ± 10.2, which was higher than that for patients with paucibacillary leprosy (2.0 ± 0.9). Despite the small number of paucibacillary patients in the study sample (n = 12), this difference was statistically significant (p = 0.002). Mean NLR (SD) in patients with a bacilloscopic index ≥3.0 was 6.2 (6.3) and was not different (p = 0.114) from that of patients with an index <3, who showed a value of 5.3 (14.2) (Table 2).

**Figure 2:**
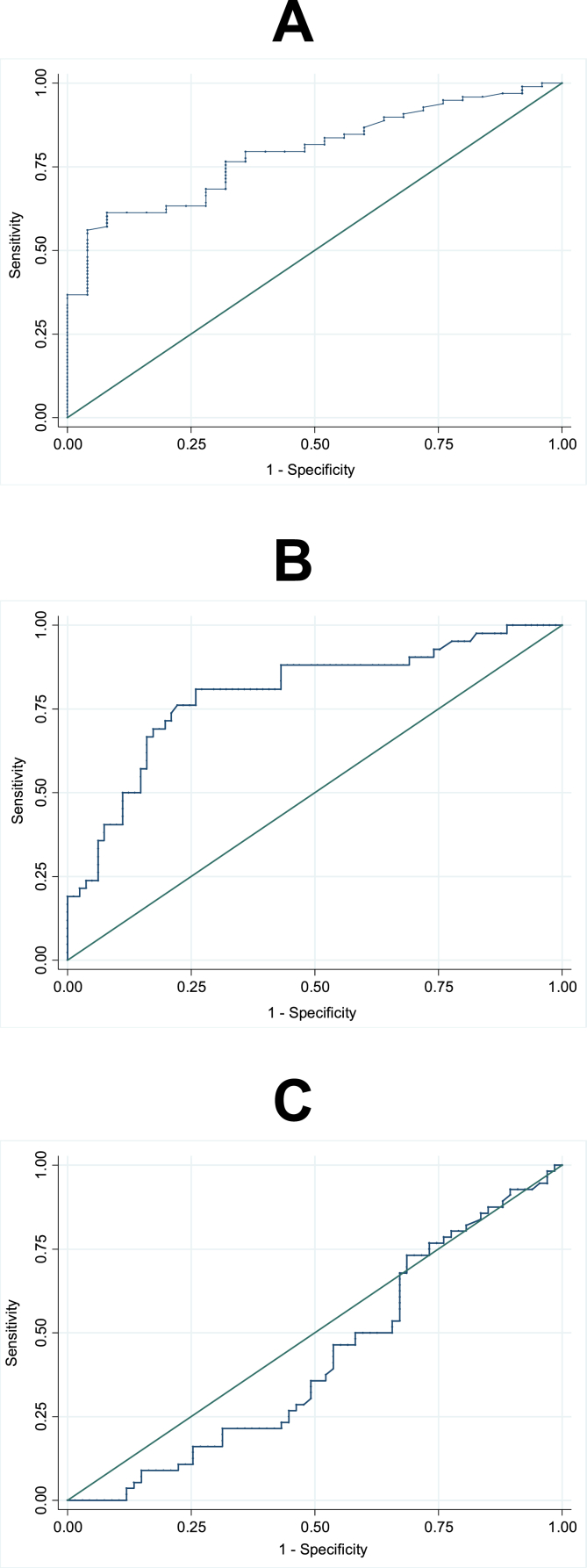
ROC curves of neutrophil-to-lymphocyte ratio for diagnosis of leprosy reaction; AUC: 0.794 (A), type 2 reaction leprosy; AUC: 0.796 (B) and type 1 reaction leprosy; AUC: 0.423 (C).

Considering all NLR values, the AUC (95% CI) was 0.794 (0.709; 0.878) for diagnosis of any leprosy reaction. AUCs (95% CI) of 0.796 (0.709; 0.883) and of 0.423 (0.321; 0.525) were found for diagnosis of type 2 reaction and type 1 reaction, respectively. The NLR cutoff point for diagnosis of any leprosy reaction was 2.75 (sensitivity 61.0%, specificity 92.0%, and accuracy 77.0%). For diagnosis of type 2 leprosy reaction, the NLR cutoff point was 2.95 (sensitivity 81.0%, specificity 74.0%, and accuracy 78.0%). For diagnosis of type 1 leprosy, NLR was not sufficiently accurate (Figure 2, A, B and C).

## 4 Discussion

This study evaluated NLR as a diagnostic biomarker of reactional states in patients with leprosy. There was a high frequency of leprosy reactions in the patients who had been referred for diagnosis and treatment of leprosy in the studied reference center in Brazil. Patients with any leprosy reaction and those with type 2 leprosy reaction had a significantly higher NLR than in patients with a type 1 leprosy reaction. NLR values of 2.75 and 2.95 were valid to significantly differentiate a patient with any leprosy reaction and those with a type 2 reaction, respectively, with high sensitivity and high specificity.

Currently, there are no laboratory biomarkers available to predict the onset of leprosy reactions among patients newly diagnosed with the infection. Early diagnosis of these reactions is not always easy and is based only on clinical characteristics of the patients. In clinical practice, the diagnosis requires observing the patient until the diagnosis becomes obvious and the treatment response demonstrates an avoidance of complications. Thus, the use of increased NLR levels in peripheral blood of patients with leprosy may represent a simple, non-invasive biomarker with high sensitivity and reasonable specificity for the safe diagnosis of leprosy reactions, especially of type 2 reaction.

The host inflammatory response plays an important role in development and progression of the leprosy reaction. Non-invasive inflammatory biomarkers, such as C- reactive protein, erythrocyte sedimentation rate, leukocyte count, procalcitonin, interferon alpha, and tumor necrosis factor α (TNF-α) have been widely used to improve diagnostic accuracy of leprosy reactions (Iyer et al., 2007; Carvalho et al., 2018). However, none of these biomarkers are useful for discriminating patients with leprosy reactions or predicting disease evolution regarding complications.

The mechanism that determines the elevation of NLR in the leprosy reaction remains uncertain. However, increasing evidence shows that leukocytes and leukocyte subtypes are classic indicators of inflammation (Horne et al., 2005) and that NLR is a biomarker of subclinical inflammation (Templeton et al., 2014)). Lymphocytes produce TNF-α, a proinflammatory cytokine that plays a main role in the innate immune response (Locksley et al., 2001). Moreover, Th17 cells, a subgroup of lymphocytes, produce interleukin 17 (IL-17), which is also produced by neutrophils (Lowes et al., 2008). Both TNF-α and IL-17 stimulate production of inflammatory mediators, which involve an increase of circulating cells. Therefore, NLR seems to adequately reflect this involvement and the systemic inflammation in patients with leprosy reaction. This concept is supported by the high number of circulating neutrophils observed in this study, although the pathogenetic mechanisms to explain this change are unclear.

In severe infections or systemic inflammation, NLR increases as a consequence of the severity of the clinical status and/or the clinical outcome of the patient (Zahorec, 2001). Recently, NLR showed greater prognostic power than that of traditional inflammatory biomarkers of infection (CRP, total leukocyte count, and neutrophil count) in youth and adults with acute community-acquired pneumonia (de Jager et al., 2012; Cataudella et al., 2017).

In the present study, the highest NLR was observed in the type 2 leprosy reaction. The interaction of neutrophils with *M. leprae* is still poorly understood, mainly due to the long incubation time (Holzer et al., 1988). However, during the type 2 leprosy reaction, cell dysfunction and elevated circulating levels of antigen-antibody immune complexes are evident (Kahawita and Lockwood, 2008). These changes are associated with increased levels of proinflammatory cytokines, and the inflammatory infiltrate is predominantly neutrophilic, accompanied by vasculitis of the subcutaneous adipose tissue and neutrophilia in the peripheral blood (Scollard et al., 2006; Kahawita and Lockwood, 2008). Therefore, this neutrophilia justifies the highest NLR mean found in patients with type 2 reactions in the current study. In contrast, changes related to neutrophils are irrelevant in type 1 leprosy reactions (Naafs and van Hees, 2016), which could explain the lack of association of NLR in this group of patients.

The diagnostic accuracy of NLR was high for diagnosis of a type 2 reaction (AUC = 0.796). This AUC value was higher than those observed in previous studies that evaluated NLR for diagnosis or prognosis of other infectious diseases (Mentis et al., 2016; Kartal and Kartal, 2017; Djordjevic et al., 2018). Therefore, it is plausible that the validity of the NLR cutoff obtained in this study was high for the diagnosis of the type 2 leprosy reaction.

Some limitations need to be recognized in the present study. A gold standard test for the diagnosis of leprosy reactions does not yet exist. In the current study, diagnosis was made based only on clinical manifestations, thus there may have been misdiagnoses. Another relevant aspect is that NLR is affected by other potential comorbidities, such as the metabolic syndrome, conditions related to inflammation, co-infections, or other factors such as nutritional status or smoking (Balta et al., 2013; Abakay et al., 2015). However, the frequency of these comorbidities was low among the patients of the study, thus this limitation did not likely affect the results. As neutrophilia and lymphocytopenia are typical phenomena of the innate immune response to various stressful insults, it is very likely that the neutrophil-to-lymphocyte ratio is increased in other acute events, making this ratio unspecific for the diagnosis of leprosy reaction. Further studies are needed to better understand the specificity of NLR for the diagnosis of the lep reaction.

In conclusion, this study constitutes a first approach to NLR as a diagnostic biomarker of leprosy reactions and compares its capacity to distinguish between type 1 *vs.* type 2 leprosy reactions using ROC curves. There is a need for additional biomarkers for the clinical diagnosis of these reactions; this need is reinforced by the high prevalence of leprosy in endemic areas that are poor around the world where health services are generally poorly structured. The discovery of simple and low-cost tools, such as NLR, could greatly increase the sensitivity of the screening and confirmation of leprosy reactions and consequently contribute to improved control of disease complications. Additional studies, especially prospective studies, are needed to confirm this conclusion.

## Declarations

### Author contribution statement

L. Gomes, C. Fontes: conceived and designed the experiments; analyzed and interpreted the data; contributed reagents, materials, analysis tools or data; wrote the paper.

Y. Morato-Conceicao: performed the experiments; analyzed and interpreted the data; contributed reagents, materials, analysis tools or data.

A. Gamabati, C. Maciel-Pereira: performed the experiments.

### Funding statement

This research was funded by a partnership fund provided by the Foundation for Research Support of the State of Mato Grosso (FAPEMAT), Brazil (FAPEMAT.0291911/2018).

### Competing interest statement

The authors declare no conflict of interest.

### Additional information

No additional information is available for this paper.

## References

[bib1] Abakay O., Abakay A., Sen H.S., Tanrikulu A.C. (2015). The relationship between inflammatory marker levels and pulmonary tuberculosis severity. Inflammation.

[bib2] Azab B., Jaglall N., Atallah J.P., Lamet A., Raja-Surya V., Farah B., Lesser M., Widmann W.D. (2011). Neutrophil-lymphocyte ratio as a predictor of adverse outcomes of acute pancreatitis. Pancreatology.

[bib3] Azab B., Bhatt V.R., Phookan J., Murukutla S., Kohn N., Terjanian T., Widmann W.D. (2012). Usefulness of the neutrophil-to-lymphocyte ratio in predicting short- and long-term mortality in breast cancer patients. Ann. Surg Oncol..

[bib4] Balta S., Demirkol S., Unlu M., Arslan Z., Celik T. (2013). Neutrophil to lymphocyte ratio may be predict of mortality in all conditions. Br. J. Cancer.

[bib5] Bantis L.E., Nakas C.T., Reiser B. (2014). Construction of confidence regions in the ROC space after the estimation of the optimal Youden index-based cut-off point. Biometrics.

[bib6] Benites-Zapata V.A., Hernandez A.V., Nagarajan V., Cauthen C.A., Starling R.C., Tang W.H. (2015). Usefulness of neutrophil-to- lymphocyte ratio in risk stratification of patients with advanced heart failure. Am. J. Cardiol..

[bib7] Bhat T., Teli S., Rijal J., Bhat H., Raza M., Khoueiry G., Meghani M., Akhtar M., Costantino T. (2013). Neutrophil to lymphocyte ratio and cardiovascular diseases: a review. Expert. Ver. Cardiovasc. Ther.

[bib8] Carvalho J.C., Araújo M.G., Coelho-Dos-Reis J.G.A., Peruhype-Magalhães V., Alvares C.C., Moreira M.L., Teixeira-Carvalho A., Martins-Filho O.A., Araújo M.S.S. (2018). Phenotypic and functional features of innate and adaptive immunity as putative biomarkers for clinical status and leprosy reactions. Microb. Pathog..

[bib9] Cataudella E., Giraffa C.M., Di Marca S., Pulvirenti A., Alaimo S., Pisano M., Terranova V., Corriere T., Ronsisvalle M.L., Di Quattro R., Stancanelli B., Giordano M., Vancheri C., Malatino L. (2017). Neutrophil-to-Lymphocyte ratio: an emerging marker predicting prognosis in elderly adults with community-acquired pneumonia. J. Am. Geriatr. Soc..

[bib10] Cedrés S., Torrejon D., Martínez A., Martinez P., Navarro A., Zamora E., Mulet- Margalef N., Felip E. (2012). Neutrophil to lymphocyte ratio (NLR) as an indicator of poor prognosis in stage IV non-small cell lung cancer. Clin. Transl. Oncol..

[bib11] de Jager C.P.C., Wever P.C., Gemen E.F.A., Kusters R., van Gageldonk-Lafeber A.B., van der Poll T., Laheij R.J.F. (2012). The neutrophil-lymphocyte count ratio in patients with community-acquired pneumonia. PLoS One.

[bib12] Dirican A., Kucukzeybek B.B., Alacacioglu A., Kucukzeybek Y., Erten C., Varol U., Somali I., Demir L., Bayoglu I.V., Yildiz Y., Akyol M., Koyuncu B., Coban E., Ulger E., Unay F.C., Tarhan M.O. (2015). Do the derived neutrophil to lymphocyte ratio and the neutrophil to lymphocyte ratio predict prognosis in breast cancer?. Int. J. Clin. Oncol..

[bib13] Djordjevic D., Rondovic G., Surbatovic M., Stanojevic I., Udovicic I., Andjelic T., Zeba S., Milosavljevic S., Stankovic N., Abazovic D., Jevdjic J., Vojvodic D. (2018). Neutrophil-to-lymphocyte ratio, monocyte-to-lymphocyte ratio, platelet-to-lymphocyte ratio, and mean platelet volume-to-platelet count ratio as biomarkers in critically ill and injured patients: which ratio to choose to predict outcome and nature of bacteremia?. Mediat. Inflamm..

[bib14] Foss N.T., Motta A.C. (2012). Leprosy, a neglected disease that causes a wide variety of clinical conditions in tropical countries. Mem. Inst. Oswaldo Cruz.

[bib15] Gomez D., Farid S., Malik H.Z., Young A.L., Toogood G.J., Lodge J.P., Prasad K.R. (2008). Preoperative neutrophil-to-lymphocyte ratio as a prognostic predictor after curative resection for hepatocellular carcinoma. World J. Surg..

[bib16] Goodman D.A., Goodman C.B., Monk J.S. (1995). Use of the neutrophil: lymphocyte ratio in the diagnosis of appendicitis. Am. Surg..

[bib17] Gupta N., Shankernarayan N.P., Dharmalingam K. (2010). *α*1-Acid glycoprotein as a putative biomarker for monitoring the development of the type II reactional stage of leprosy. J. Med. Microbiol..

[bib18] Holzer T.J., Kizlaitis L., Vachula M., Weaver C.W., Andersen B.R. (1988). Human phagocytic cell responses to Mycobacterium leprae and Mycobacterium bovis Bacillus Calmette-Guerin. An in vitro comparison of leprosy vaccine components. J. Immunol..

[bib19] Horne B.D., Anderson J.L., John J.M., Weaver A., Bair T.L., Jensen K.R., Renlund D.G., Muhlestein J.B. (2005). Which white blood cell subtypes predict increased cardiovascular risk?. J. Am. Coll. Cardiol..

[bib20] Iyer A., Hatta M., Usman R., Luiten S., Oskam L., Faber W., Geluk A., Das P. (2007). Serum levels of interferon-gamma, tumour necrosis factor-alpha, soluble interleukin-6R and soluble cell activation markers for monitoring response to treatment of leprosy reactions. Clin. Exp. Immunol..

[bib21] Jacobson R.R., Krahenbuhl J.L. (1999). Leprosy. Lancet.

[bib22] Kahawita I.P., Lockwood D.N. (2008). Towards understanding the pathology of erythema nodosum leprosy. Trans. R. Soc. Trop. Med. Hyg..

[bib23] Kartal O., Kartal A.T. (2017). Value of neutrophil to lymphocyte and platelet to lymphocyte ratios in pneumonia. Bratisl. Lek. Listy.

[bib24] Locksley R.M., Killeen N., Lenardo M.J. (2001). The TNF and TNF receptor superfamilies: integrating mammalian biology. Cell.

[bib25] Lockwood D.N., Saunderson P. (2012). Nerve damage in Leprosy: a continuing challenge for scientists, clinicians and service providers. Int. Health.

[bib26] Loonen A.J.M., de Jager C.P.C., Tosserams J., Kusters R., Hilbink M., Wever P.C., van den Brule A.J.C. (2014). Biomarkers and molecular analysis to improve bloodstream infection diagnostics in an emergency care unit. PLoS One.

[bib27] Lowes M.A., Kikuchi T., Fuentes-Duculan J., Cardinale I., Zaba L.C., Haider A.S., Bowman E.P., Kruegere J.G. (2008). Psoriasis vulgaris lesions contain discrete populations of Th1 and Th17 T cells. J. Invest. Dermatol..

[bib29] Mentis A.F., Kyprianou M.A., Xirogianni A., Kesanopoulos K., Tzanakaki G. (2016). Neutrophil-to-lymphocyte ratio in the differential diagnosis of acute bacterial meningitis. Eur. J. Clin. Microbiol. Infect. Dis..

[bib30] Naafs B., van Hees C.L. (2016). Leprosy type 1 reaction (formerly reversal reaction). Clin. Dermatol..

[bib31] Nath I., Saini C., Valluri V.L. (2015). Immunology of leprosy and diagnostic challenges. Clin. Dermatol..

[bib32] Papa A., Emdin M., Passino C., Michelassi C., Battaglia D., Cocci F. (2008). Predictive value of elevated neutrophil-lymphocyte ratio on cardiac mortality in patients with stable coronary artery disease. Clin. Chim. Acta.

[bib33] Raffe S.F., Thapa M., Khadge S., Tamang K., Hagge D., Lockwood D.N. (2013). Diagnosis and treatment of leprosy reactions in integrated services - the patients’ perspective in Nepal. PLoS Negl. Trop. Dis..

[bib34] Riley L.K., Rupert J. (2015). Evaluation of patients with leukocytosis. Am. Fam. Phys..

[bib35] Scollard D., Adams L.B., Gillis T.P., Krahenbuhl J.L., Truman R.W., Williams D.L. (2006). The continuing challenges of leprosy. Clin. Microbiol. Rev..

[bib36] Templeton A.J., McNamara M.G., Šeruga B., Vera-Badillo F.E., Aneja P., Ocana A., Leibowitz-Amit R., Sonpavde G., Knox J.J., Tran B., Tannock I.F., Amir E. (2014). Prognostic role of neutrophil-tolymphocyte ratio in solid tumors: a systematic review and metaanalysis. J. Natl. Cancer Inst..

[bib37] Torun S., Tunc B.D., Suvak B., Yildiz H., Tas A., Sayilir A., Ozderin Y.O., Beyazit Y., Kayacetin E. (2012). Assessment of neutrophil-lymphocyte ratio in ulcerative colitis: a promising marker in predicting disease severity. Clin. Res. Hepatol. Gastroenterol..

[bib38] WHO. World Health Organization (2016). Global Leprosy Strategy 2016-2020: Accelerating towards a Leprosy-free World.

[bib39] WHO. World Health Organization (2012). WHO expert committee on leprosy. World Health Organ. Tech. Rep. Ser..

[bib41] Yamanaka T., Matsumoto S., Teramukai S., Ishiwata R., Nagai Y., Fukushima M. (2007). The baseline ratio of neutrophils to lymphocytes is associated with patient prognosis in advanced gastric cancer. Oncology.

[bib42] Zahorec R. (2001). Ratio of neutrophil to lymphocyte counts-rapid and simple parameter of systemic inflammation and stress in critically ill. Bratisl. Lek. Listy.

